# Development of Novel Residual-Dense-Attention (RDA) U-Net Network Architecture for Hepatocellular Carcinoma Segmentation

**DOI:** 10.3390/diagnostics12081916

**Published:** 2022-08-08

**Authors:** Wen-Fan Chen, Hsin-You Ou, Han-Yu Lin, Chia-Po Wei, Chien-Chang Liao, Yu-Fan Cheng, Cheng-Tang Pan

**Affiliations:** 1 Institute of Medical Science and Technology, National Sun Yat-sen University, Kaohsiung 80424, Taiwan; 2 Liver Transplantation Program and Department of Diagnostic Radiology, and Surgery Kaohsiung Chang Gung Memorial Hospital, and Chang Gung University College of Medicine, Kaohsiung 83301, Taiwan; 3 Department of Mechanical and Electro-Mechanical Engineering, National Sun Yat-sen University, Kaohsiung 80424, Taiwan; 4 Department of Electrical Engineering, National Sun Yat-sen University, Kaohsiung 80424, Taiwan; 5 Institute of Advanced Semiconductor Packaging and Testing, College of Semiconductor and Advanced Technology Research, National Sun Yat-sen University, Kaohsiung 80424, Taiwan

**Keywords:** computed tomography, hepatocellular carcinoma, attention U-Net, ResNet, DenseNet, staging classification

## Abstract

The research was based on the image recognition technology of artificial intelligence, which is expected to assist physicians in making correct decisions through deep learning. The liver dataset used in this study was derived from the open source website (LiTS) and the data provided by the Kaohsiung Chang Gung Memorial Hospital. CT images were used for organ recognition and lesion segmentation; the proposed Residual-Dense-Attention (RDA) U-Net can achieve high accuracy without the use of contrast. In this study, U-Net neural network was used to combine ResBlock in ResNet with Dense Block in DenseNet in the coder part, allowing the training to maintain the parameters while reducing the overall recognition computation time. The decoder was equipped with Attention Gates to suppress the irrelevant areas of the image while focusing on the significant features. The RDA model was used to identify and segment liver organs and lesions from CT images of the abdominal cavity, and excellent segmentation was achieved for the liver located on the left side, right side, near the heart, and near the lower abdomen with other organs. Better recognition was also achieved for large, small, and single and multiple lesions. The study was able to reduce the overall computation time by about 28% compared to other convolutions, and the accuracy of liver and lesion segmentation reached 96% and 94.8%, with IoU values of 89.5% and 87%, and AVGDIST of 0.28 and 0.80, respectively.

## 1. Introduction

With the change of lifestyle, alcoholic hepatitis and nonalcoholic steatohepatitis increased in Taiwan. Hepatitis B, hepatitis C, alcoholic hepatitis and nonalcoholic steatohepatitis often evolve from the above chronic hepatitis to cirrhosis and eventually to liver cancer [1]. Liver cancer ranked second among the top 10 causes of cancer deaths in Taiwan in 2020 [2]. Early diagnosis of liver cancer can increase the survival rate. However, physicians can only determine the stage of cancer by carefully examining the results of magnetic resonance imaging (MRI) and computed tomography (CT), which may lead to the possibility of incorrect judgment. Most liver tumors require tumor biopsy to confirm the diagnosis, which is currently the most accurate method. However, tumor biopsy is an invasive test and has unavoidable complications such as bleeding and needle tract tumor seeding. In addition, the abdominal cavity has multiple organs that cannot be examined for cancer using only abdominal ultrasound, but need to be combined with CT, blood sampling and biopsy. Computed tomography can provide continuous images in different directions for more accurate cancer diagnosis [3]. If this method is trained with deep learning (DL), it would be able to help physicians to achieve better results in cancer treatment and prevention, as well as reduce the error rate of manual image recognition and the need for tissue biopsy when the imaging cannot be manually recognized.

Compared to MRI, CT is more accurate for organ detection and arterial, venous, and delayed phases. Further, CT images of tumors that cannot be identified by the naked eye are usually used with contrast [4]. With the increase in the aging population and modern medical advances, the number of patients being tested has increased, and the corresponding volume of imaging data has increased exponentially, leading to a dramatic increase in the workload of physicians, examiners, and radiologists. As a result, DL can allow physicians to reduce the amount of time needed to examine image data. When DL is used in visual processing, it is necessary to go through image pre-processing and image annotation (ground truth) steps. Further, when DL is applied to medical image recognition [5], it is difficult to identify the CT image itself. In order to ensure the benchmark of the model training, it is necessary to use the assistance and confirmation of the professional physician for the labeling of the lesion. In this study, the files used were images without contrast medium, and the image contrast was improved by image processing and image equalization to achieve the same image as the image with contrast medium, which facilitated the overall recognition result.

Deep learning (DL) is an evolution of artificial intelligence (AI) and machine learning (ML), which is designed to emulate the way the human brain thinks, with the expectation that the machine would have the ability to learn as humans do. The goal of AI is to enable computers to have the same range of cognitive capabilities as humans [6], while ML uses large amounts of data and algorithms for training to generate models [7,8,9,10,11]. For DL, it is expected to simulate the operation of neural networks formed by neurons in the human brain [12,13] for applications in visual recognition [14,15], speech recognition, and biomedicine. The artificial neural network (ANN) used in DL is so called because its structure resembles human neural transmission patterns, and deep learning uses training images in the same way as human visual observation, to provide images that are easier to distinguish and achieve better recognition results. Further, among many neural networks, the convolutional neural network (CNN) is one of the most representative networks for medical image related processing [16,17,18,19,20]. Initially, the CNN for simple recognition of handwritten characters was proposed by LeCun et al. [21], and was re-proposed in 1998 with the LeNet-5 network architecture for handwritten digit recognition [22]. Then the U-Net was proposed by Ronneberger et al. [23] in 2015 to solve the problem of medical image segmentation; thus, the U-shaped structure of the U-Net is better used for the skip connection structure. Another feature of U-Net convolution is that it can use an encoder to extract features to reduce the output size, and then restore them to the original size by a decoder, which can effectively retain more information of the original image.

In this study, the U-Net was used as the basis and the convolution was modified. The encoder was integrated with the concept of residual block (ResBlock) in deep residual neural network (ResNet) proposed by He et al. [24] in 2015 and the dense convolutional neural network (DenseNet) proposed by Huang et al. [25] in 2018, which connects each layer to every other layer in a feed-forward fashion. In dense block (DB), the Attention Dense-U-Net proposed by Li Shuyi et al. [26] in 2019 was utilized. Due to the complexity of this network structure, it takes more time to update the network parameters during the training process. In the literature, it was chosen to increase the appropriate time cost to improve the segmentation accuracy of the lesion. In the decoder part, the attention gates (AGs) module in the Attention U-Net model proposed by Oktay et al. [27] in 2018 was utilized and applied to medical images. It is known that the model trained using AGs is capable of suppressing irrelevant regions in the image while focusing on useful salient features. Therefore, this study combined the above algorithms and proposed the Residual-Dense-Attention (RDA) U-Net model. This convolutional neural network (CNN) model was trained from CT images to perform the segmentation between liver organs and lesions.

## 2. Proposed Method

### 2.1. Data Set and Image Pre-Processing

**Figure 1:**
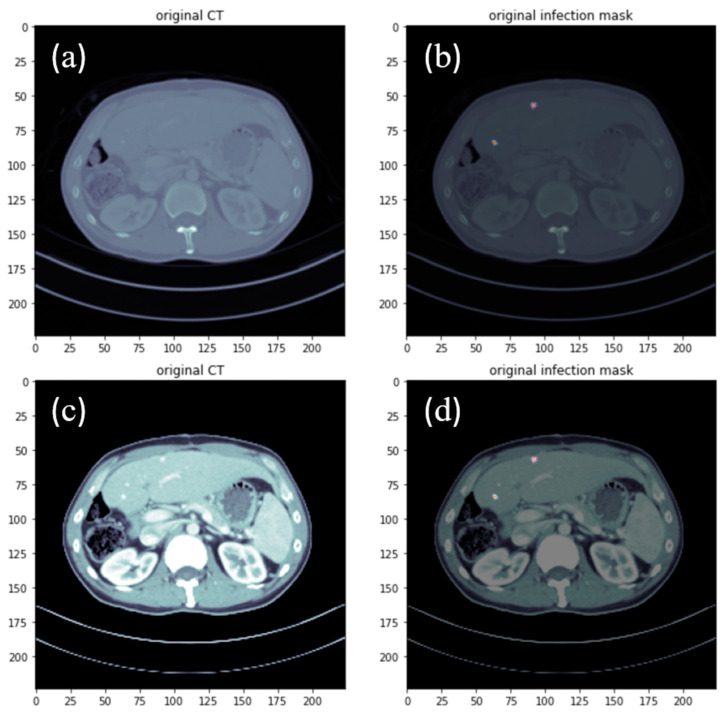
Before adjustment of HU values from the (**a**) original hospital CT image data and (**b**) annotated lesion image data (white colored is the location of the lesion); after adjustment of HU values from the (**c**) original hospital CT image data and (**d**) annotated lesion image data (white colored is the location of the lesion).

Due to the small amount of data in medical images and the difficulty of accessing them, it is necessary to obtain data by data enhancement [28]. In this study, a mixture of open source data and hospital data was used to increase the diversity of the trained images in addition to the training volume. The data from the Internet were filtered and made public before they can be used. The current open source data are broadly divided into two types, one is a clear liver without atrophic margins and clear lesions, and the second is a normal liver without cancer, with these two types accounting for the majority of training. In this study, CT images of 500 patients were provided by Kaohsiung Chang Gung Memorial Hospital, and a variety of lesion files were selected for training (such as patients with large tumors, small tumors, single tumors, multiple tumors, and liver atrophy). After filtering to 100 patients, files with very small lesions, interference in the periphery of the images, and extremely difficult to identify areas were deducted, and finally 65 patients were used for training. The medical images were in DICOM format. For post-training and conversion of the trained images into 3D perspective, an MRI converter [29] was used to convert the image format to Nifti files while preserving the original data (such as thickness per layer and spatial location). Since medical images of lesions are difficult to identify without contrast, the Hounsfield scale, which contains Hounsfield unit (HU) information, was adjusted by pre-processing to improve image clarity [30]. The image before and after adjustment is shown in Figure 1. As can be seen in the figure, both lesions appeared white after adjustment. In order to clearly show the lesions, the original CT image and the Ground Truth were overlaid by the code for correct viewing; hence, the white or red border color was displayed by the dazzling border.

### 2.2. Designed Model Framework

U-Net can be divided into two parts: encoder and decoder. The structure on the left side of the framework is an encoder for feature interception, and a copy and crop (also known as skip connection) splicing structure was used between the encoder and decoder to integrate the feature information between them. The unique part of the designed model was that the decoder used the up-convolution method, where the features were linear and gradually increase in size. Further, the image size at the output was the same as the image size at the input, while the information at the input gradually decreased in a conventional convolution structure. As a result, the use of up-convolution can overcome the loss problem that occurs during feature transfer from the encoder.

**Figure 2:**
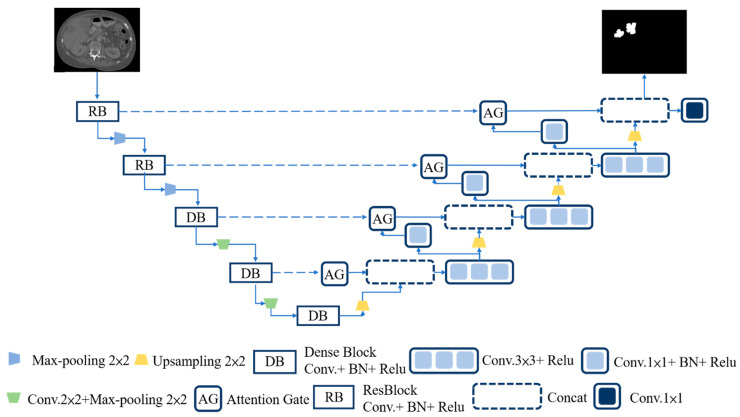
Designed model framework.

The U-Net architecture has the identical image size on the input side and the output side, which is a promising application in medical imaging. An encoder can be used to extract the features to reduce the output size, and then restore to the original image size by the decoder to retain more original image information. As a result, U-Net was used as the basis for convolutional modification in this study, and the overall framework of the model is shown in Figure 2.

### 2.3. Model Encoder

**Figure 3:**
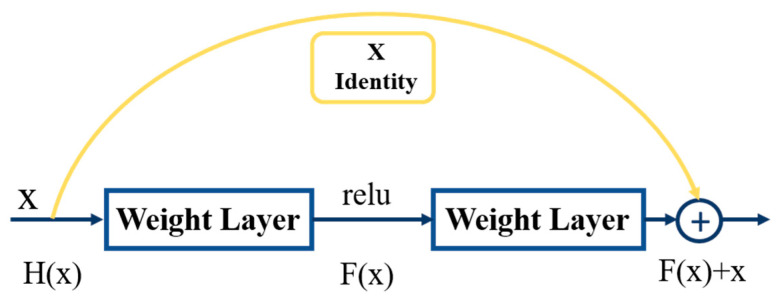
Structure of ResBlock module.

ResBlock was used to connect the input side to the output side through shortcut, which was equivalent to an equal mapping across the intermediate layers. The above approach does not generate additional parameters and increase the complexity of the computation, while at the same time it ensures that the performance after deepening the network is not worse than before, as shown in Figure 3. If the original output is H(x) and the optimized output is x at the input, the expectation is H(x) = x, and the equation F(x) = H(x) − x is optimized to be close to zero by ResBlock. The use of residuals on the ResBlock can mitigate the problem of gradient disappearance in the overall neural network, which is equivalent to crossing the intermediate layers and performing the addition of the connected layers. Performing an equivalent mapping does not generate additional parameters and increase the computational complexity, but the skipped approach used in this residual block to retain features may limit the performance of the network [31,32], indicating that this shortcut may lead to area problems and reduce the network learning capability [33].

**Figure 4:**
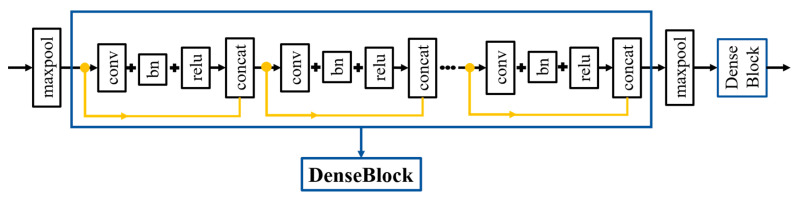
Detailed structure of DenseBlock.

In order to reduce the training time of the overall model, DenseBlock was used to chain the feature maps through concatenation, and the feature maps were merged in a dimensional way (i.e., feature reuse), which can reduce the number of feature maps on the input side. This method can not only reduce the amount of calculation, but also fuse the features of each channel, which is equivalent to connecting each layer directly with input and loss to reduce the disappearance of gradient, as shown in Figure 4.

### 2.4. Model Decoder

**Figure 5:**
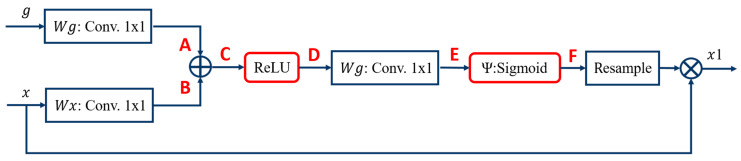
Details of AGs module.

The use of the Attention module in the image caption domain was published by Xu et al. [34] in 2015. Attention mechanisms can be divided into two types, namely hard attention and soft attention. Hard attention is mainly used in region iteration and cropping, which is not differentiable; thus, the model is not easy to train and needs to be optimized by strengthening the learning parameters. In contrast, soft attention is differentiable, so that gradients can be computed through neural networks, and weights can be learned by forward propagation and backward feedback. In the right half of the model (shown in Figure 2), where the decoder was located, the AGs module is further explained, as shown in Figure 5. Models trained with AGs would suppress irrelevant areas of the image while focusing on useful salient features, similar to the concept of human visual attention. Human vision can focus on specific points or areas while suppressing surrounding areas. The results showed that the use of AGs can maintain the computational efficiency as well as improve the predictive performance of U-Net under different data and training. When an AGs module was used in the U-Net architecture, a skip connection was applied in front of the encoder on the input side for each resolution feature and the decoder on the output side for the splicing of the corresponding feature to a significant feature. By skipping irrelevant areas of the connection, the training accuracy problems caused by the noise component were eliminated.

The details of the AGs module are as follows: *g* was obtained from A by the convolution of *Wg*, and *x* was obtained from B by the convolution of *Wx*. The g came from the lower layer of *x*, and the two images had different dimensions; thus, the *x* had to be down-sampled first, and then the two images were overlapped to obtain C. Further, the C part overlapped the A and B images, and then linearly transformed the excitation functions such as ReLU, Ψ, and Sigmoid into D and F to mitigate the overfitting of the network.

## 3. CT Images of the Liver

The CT images used in this study consisted of the LiTS dataset [35], a web-based open source data set provided by medical institutions worldwide, which contained CT images of 131 patients. A total of 43 patients were selected from the LiTS dataset, including 28 patients with a left-sided liver and 15 patients with a right-sided liver, and 48 patients from Kaohsiung Chang Gung Memorial Hospital (KCGMH) were trained to form a dataset with a total of 91 patients. This dataset was converted into a total of 19,514 images, which were divided into a training set and a validation set according to the ratio of 8:2. In order to accommodate the model size and to avoid exceeding the GPU memory, the original 512 × 512 pixel-valued images were reduced to 224 × 224 to improve the computational efficiency.

## 4. Results and Discussion

### 4.1. Training Environment and Parameter Setting

The original CT, liver region mask, and tumor location mask were used for each training image set. The parameters of the convolutional network used in this study were set to 100 and 8 for epoch and batch, respectively. The initial learning rate was set to 10^−6^, and the ADAM optimizer was used to update the parameters of the overall network. During the training process, the images were randomly assigned to 80% for training set and 20% for verification set, which was the optimal allocation to achieve the best training results. The completed training network was further tested on the new data to confirm the test results. The models were trained and tested in the following environments: Intel^®^ Core™ i7-9700KF CPU, RTX 2080 Ti GPU, DDR4 128GB memory, and Windows 10. The software was used with Keras 2.3.0, Tensorflow-gpu1.14, and Python 3.7.7.

### 4.2. Evaluation Metrics

The performance of the proposed model was evaluated using the following metrics: Accuracy (ACC), Dice coefficient, Intersection over Union (IoU), and Average Hausdorff Distance (AVGDIST) [36]. Those metrics could be computed by four measures: *TP* (true positive), *TN* (true negative), *FP* (false positive), and *FN* (false negative). Since the accuracy is calculated by taking the TN and the black part without lesions or liver, the result of accuracy is generally better than other calculation methods (IoU, DSC, and AVGDIST). For more detailed description, TN adds the correct black part to the calculation, while the black part in Ground Truth accounts for the majority. The accuracy is expressed by:

(1) $$\mathrm{Accuracy}\;=\;\frac{TP+TN}{TN+FP+TP+FN}.$$

The standard for image segmentation is often calculated using the Dice coefficient (DSC), as shown in Equation (2), which indicates the similarity of two samples in the range of 0 to 1. The best result for segmentation is 1 and the worst value is 0.

(2) $$\mathrm{DSC}\;=\;\frac{2TP}{2TP+FP+FN}.$$

IoU is to divide the prediction result into 0 for the black part and 1 for the white part. Then, the prediction result will be overlapped with Ground Truth, and the intersection and union of the overlapped images will be calculated. The IoU formula calculated for medical images with more black areas is as follows:

(3) $$\mathrm{IoU}\;=\;\frac{TP}{FP+TP+FN},$$

The Hausdorff Distance is sensitive to the segmented boundary range. This equation can compare the boundary range distance between the training result and the actual result of the image, and thus is mainly used for image segmentation. The formula is shown below:

(4) $$\mathrm{Average}\;\mathrm{Hausdorff}\;\mathrm{Distance}\;=\;\left(\frac{GtoS}{G}+\frac{StoG}{S}\right)/2$$

### 4.3. Comparison of Training Time and Convolution Parameters

In Attention Dense-U-Net proposed by Li et al. [26] in 2019, the associated network structure was more complex, and thus more time was required to update the network parameters during the training process. The work mainly focused on increasing the time cost in exchange for improving the accuracy of tumor segmentation. In our study, the designed convolutional network can achieve the accuracy of the network with less time cost compared to the Attention U-Net and Attention Dense-U-Net convolutional networks. Further, the convolutional parameters of the designed model are higher (total value) than those of the Attention Res-UNet. By hopping connection of ResBlock, the overall network training time can be reduced, and the accuracy of the overall model convolutional parameters can be maintained by DenseBlock. A comparison of the model parameters and training time is shown in Table 1.

### 4.4. Identification Results of Liver and Lesions

The performance of segmentation was compared using the above indicative evaluation for our convolution and the three associated convolutions namely Attention U-Net [27], Attention Dense-U-Net [28], and Attention Res-UNet [37]. The evaluation results of ACC, DSC, IoU and AVGDIST for liver segmentation are presented in Table 2. In terms of ACC, since the ACC method mentioned above includes the accurate black part in a large area compared to other methods, the result of the ACC calculation is better. Further, comparing the other computation methods, it can be observed that the proposed convolution (RDA U-Net) gives better results in DSC and IoU computations than Attention U-Net and Attention Res-UNet, and the calculation of AVGDIST for distance is also more accurate. In terms of Attention Dense-U-Net, due to the fully connected approach, it has more feature reuse than other convolutions, resulting in more GPU memory and computation time required to obtain higher ACC, DSC, IoU, and AVGDIST. That is, although the overall results of Attention Dense-U-Net are the best, the computation time of our proposed convolution (RDA U-Net) can be reduced by 28% and meanwhile good computation results can also be obtained.

The evaluation results of ACC, DSC, IoU, and AVGDIST for lesion segmentation are presented in Table 3. It can be found that although our convolution was slightly lower than Attention Dense-U-Net in the ACC, DSC, and IoU, the Attention Dense-U-Net took 607 s to train one turn of liver image [26] while our proposed convolution (RDA U-Net) only took 437 s. After comparing the training time and the results, it is believed that the results obtained using our proposed convolutional network (RDA U-Net) are in a good range. Specifically, for the distance comparison of AVGDIST, it was observed that our convolution results were higher than the Attention Res-UNet results for liver segmentation due to the fact that the Attention Res-UNet uses a hopping connection, which would lead to poor learning results for small lesions. The average distance was calculated by overlaying the prediction results with Ground Truth and then calculating the distance between the two edges. If the distance between the two is closer, there is a lower value, which means a more accurate prediction. In this case, our proposed convolutional training distance of 0.8058 is better than the Attention Res-UNet value of 9.3871, which indicates that Attention Res-UNet is not suitable for lesion segmentation.

**Figure 6:**
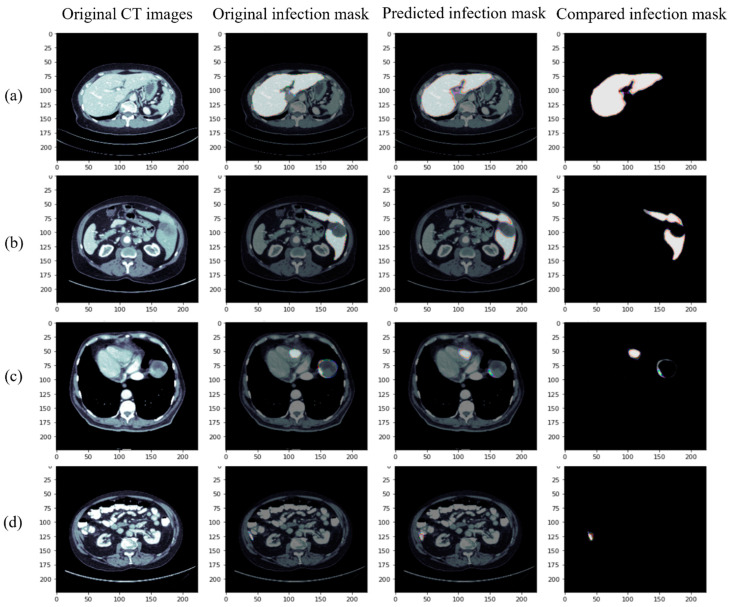
Segmentation results of our proposed model for the liver: (**a**) normal liver, (**b**) right side of the liver, (**c**) liver near the heart, and (**d**) liver near the lower abdomen (from left to right, original CT image, Ground Truth, predicted results of our proposed model, and superposition of Ground Truth and predicted results).

The segmentation results of our proposed model (RDA U-Net) after training on the liver are shown in Figure 6. It can be observed that both the normal liver (Figure 6a) and the patient with the liver located on the right side (Figure 6b) can be accurately fit for segmentation. Further, although the liver near the heart position (Figure 6c) was successfully captured, the small segmentation on its right side was not completely captured. The location of the liver can be ideally distinguished and segmented (Figure 6d), where there are many organs close to the lower abdomen, and the results showed that our proposed model (RDA U-Net) has a good segmentation effect on the location of the liver.

**Figure 7:**
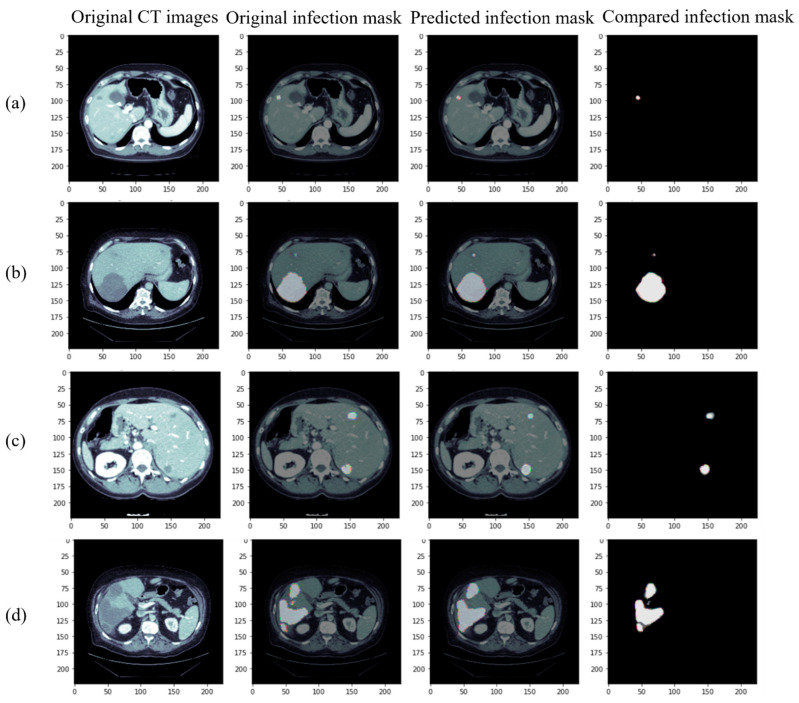
Segmentation results of our proposed model for the lesion: (**a**) small tumor (**b**) large tumor (with small tumor) (**c**) multiple small tumors (**d**) multiple large tumors (from left to right, original CT image, Ground Truth, predicted results of our proposed model, and superposition of Ground Truth and predicted results).

Figure 7 shows the segmentation results of our proposed model (RDA U-Net) after training at the lesion site. It can be seen that both small tumors (Figure 7a) and large tumors (Figure 7b) can be successfully captured, even for those small tumors contained in them. Further, the ideal segmentation results were also obtained for the locations of multiple small tumors in Figure 7c. Several large tumors in Figure 7d were successfully captured, but the separate tumors in the image could not be fully captured. For the subsequent training, more lesions with irregular margins can be used for training to improve the results.

**Figure 8:**
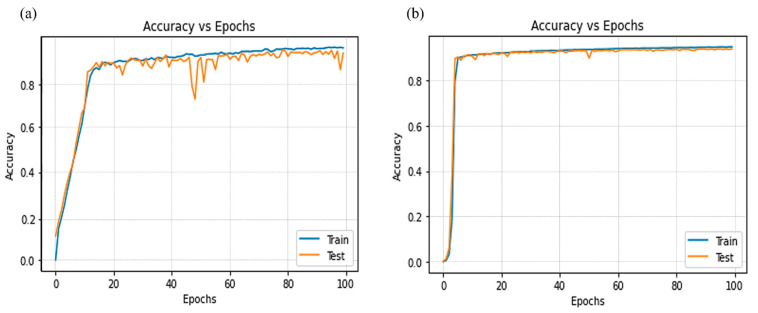
Results of training in 91 patients with mixed LiTS and KCGMH: (**a**) liver and (**b**) lesions.

Figure 8 shows the training results of 91 patients with mixed LiTS and KCGMH, for liver and lesion, respectively. A total of 19,514 images were used with image pre-processing to convert image values from 512 × 512 to 224 × 224 pixel values and also restricted HU values. Then, our convolutional RDA U-Net with optimized learning rate was used for random mixing and subsequent training of 100 epochs. It can be found that excellent learning results (curve fitting) were obtained with ACC values of 0.9600 for the liver and 0.9477 for the lesion.

**Figure 9:**
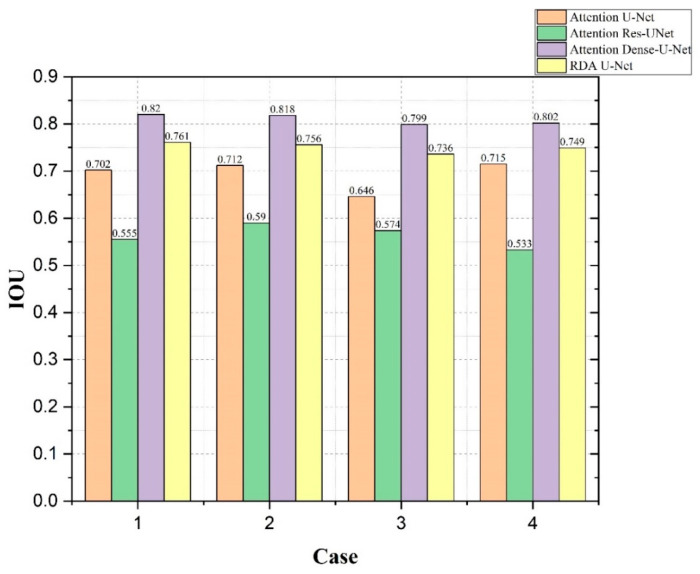
Comparison of IoU values using Attention U-Net, Attention Res-UNet, Attention Dense-U-Net and our RDA U-Net for each of the 4 cases.

**Figure 10:**
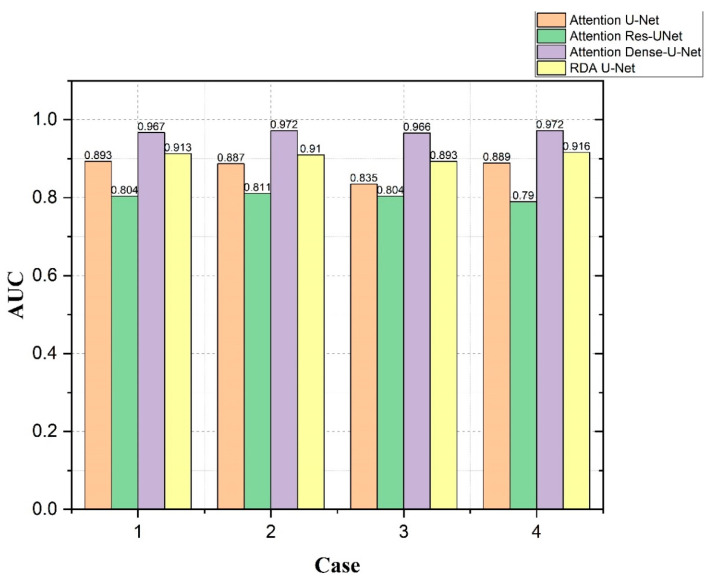
Comparison of ROC values using Attention U-Net, Attention Res-UNet, Attention Dense-U-Net and our RDA U-Net for each of the 4 cases.

Figure 9 and Figure 10 show the IoU and AUC values for the four cases using Attention U-Net, Attention Res-UNet, Attention Dense-U-Net and our RDA U-Net. For the four cases in Figure 9, our convolutional network was only slightly lower than Attention Dense-U-Net, and the results were better than the other two convolutional networks. Further, the segmentation of small tumors and multiple small tumors was better than the Case 3 of Attention U-Net and the Case 4 of Attention Res-UNet, respectively. The AUC in Figure 10 is the area calculated under the ROC curve. When the AUC value is higher, which implies that the area under the ROC is larger, and the curve is closer to the upper left, this indicates better performance. From both results, it can be observed that our convolutional RDA U-Net maintained better segmentation performance for the four cases.

**Figure 11:**
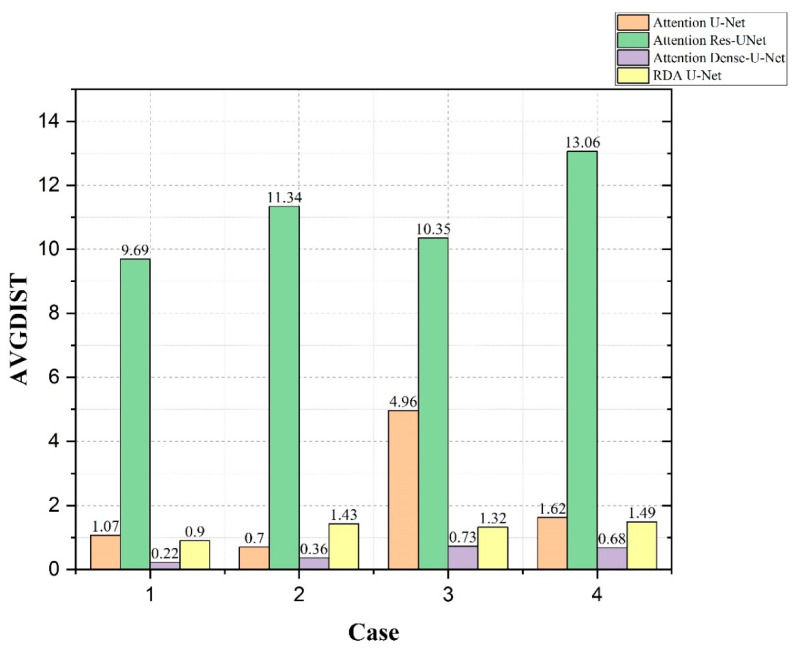
Comparison of AVGDIST values using Attention U-Net, Attention Res-UNet, Attention Dense-U-Net and our RDA U-Net for each of the 4 cases.

In Figure 11, the AVGDIST, also known as average distance between, was compared for four cases using Attention U-Net, Attention Res-UNet, Attention Dense-U-Net, and our RDA U-Net. The calculation was based on the average distance between Ground Truth and the edge of the predicted result; thus the lower the value, the better the result. The accuracy, IoU and AUC results of Attention Res-UNet were not much different from the others as shown in Table 3, Figure 9 and Figure 10. However, the results in Figure 11 showed that the discrimination results of Attention Res-UNet for small tumors were much worse than the others. Although the results for multiple mixed tumors in Case 2 were slightly higher than those for Attention U-Net, the results for single small tumors in Case 3 showed that our RDA U-Net convolutions were better than those for Attention U-Net convolutions.

## 5. Conclusions

With the advancement of medical science and technology, imaging examinations nowadays account for a large proportion of the total number of examinations. Trained models can reduce the labor cost through the artificial intelligence of image recognition and the assistance of professional physicians in liver and lesion delineation. In this paper, RDA U-Net model architecture was proposed for the automatic segmentation of lesions in CT images. This model used ResBlock and DenseBlock in the coder so that the overall network had sufficient feature images and parameters without requiring long computation time. In the decoder part, Attention Gates was used to improve the prediction performance under different data and training by helping the model to suppress image irrelevant regions while focusing on useful salient features. The results of CT image training for 91 patients using mixed LiTS and KCGMH showed ACC values of 0.9600 and 0.9477 for the liver and lesions, respectively, with an overall reduction in computation time of about 28% compared to other convolutions. The IoU values were 89.5% and 87%, and the AVGDIST values were as low as 0.28 and 0.80, respectively. From the results, it is clear that the accuracy of our proposed convolution was higher compared to other convolutions, although it was slightly lower compared to the Attention Dense-U-Net value; however, less time was used to complete the training and obtain the approximate accuracy.

## Figures and Tables

**Table 1:** Comparison of model parameters and training time.

Method	Parameter	Training Time for Tumor (s)	Training Time for Liver (s)
**Proposed Work (RDA U-Net)**	13,053,861	437	375
**Attention U-Net [27]**	35,238,293	615	530
**Attention Dense-U-Net [26]**	14,374,021	607	556
**Attention Res-UNet [37]**	12,981,573	408	360

**Table 2:** Results of different models for evaluation of liver segmentation.

Method	ACC	DSC	IoU	AVGDIST
**Proposed Work (RDA U-Net)**	0.9600	0.8945	0.8113	0.2723
**Attention U-Net [27]**	0.9030	0.8437	0.7466	0.4141
**Attention Dense-U-Net [26]**	0.9721	0.9021	0.8236	0.2120
**Attention Res-UNet [37]**	0.9709	0.8696	0.7729	0.3427

**Table 3:** Results of different models for evaluation lesion segmentation.

Method	ACC	DSC	IoU	AVGDIST
**Proposed Work (RDA U-Net)**	0.9477	0.8703	0.7714	0.8058
**Attention U-Net [27]**	0.9227	0.7588	0.6187	1.2789
**Attention Dense-U-Net [26]**	0.9582	0.9011	0.8204	0.3429
**Attention Res-UNet [37]**	0.9323	0.7310	0.6245	9.3871

## Data Availability

Restrictions apply to the availability of these data. Data was obtained from Kaohsiung Chang Gung Memorial Hospital and are available H.-Y.O., C.-C.L. and Y.-F.C. with the permission of Kaohsiung Chang Gung Memorial Hospital, Taiwan.
